# Supplementary material for: Electron-mediated control of nanoporosity for targeted molecular separation in carbon membranes
Source: Nat Commun. 2022 Aug 24;13:4972. doi: 10.1038/s41467-022-32703-4 (PMC9402951; doi:10.1038/s41467-022-32703-4)
Supplement: Supplementary file 1 — Supporting Information [file 41467_2022_32703_MOESM1_ESM.pdf]

Supporting Information

Electron-mediated Control of Nanoporosity for Targeted Molecular Separation in Carbon membranes

Banseok Oh[1], Hyeokjun Seo[1], Jihoon Choi[1], Sunggyu Lee[1], Dong-Yeun Koh[1,2]*

Affiliations

[1] Department of Chemical and Biomolecular Engineering, Korea Advanced Institute of Science and Technology, Daejeon 34141, Republic of Korea

[2] KAIST Institute for NanoCentury, Daejeon 34141, Republic of Korea

*Corresponding author E-mail: dongyeunkoh@kaist.ac.kr (D.-Y. Koh)

Table of contents

Supplementary Note 1. Fickian transport diffusion coefficient determination.

Supplementary Note 2. Estimation apparent activation energy of permeation using Arrhenius regression

Supplementary Fig. 1. Chemical structure of polyimide 6FDA:BPDA(1:1)-DAM (X=1, Y=1).

Supplementary Fig. 2. Oxygen line scan from Energy-dispersive X-ray spectrometer (EDS)

Supplementary Fig. 3. Grazing-incidence wide-angle X-ray scattering (GI-WAXS) 2D scattering image.

Supplementary Fig. 4. X-ray photoelectron spectroscopy (XPS) survey scan.

Supplementary Fig. 5. X-ray photoelectron spectroscopy (XPS) depth profiling.

Supplementary Fig. 6. Oxidized carbon layer thickness as irradiation dosage.

Supplementary Fig. 7. Results of Raman spectroscopy.

Supplementary Fig. 8. Schematic representation of constant-volume gas permeation apparatus.

Supplementary Fig. 9. Separation performance of the prepared CMS membranes in He, H_2 and CO_2.

Supplementary Fig. 10. Schematic for selective gas permeation in CMS.

Supplementary Fig. 11. Separation performance of precursor and CMS membranes.

Supplementary Fig. 12. Gas permeation data of highly irradiated CMS membranes.

Supplementary Fig. 13. Permeation data of 600 °C CMS membranes including 500 °C data.

Supplementary Fig. 14. Custom-built pressure decay system illustration.

Supplementary Fig. 15. C_2H_4 and C_2H_6 pressure decay sorption curves at fugacity 0.1.

Supplementary Fig. 16. C_2H_4 and C_2H_6 pressure decay sorption curves at fugacity 0.3.

Supplementary Fig. 17. C_2H_4 and C_2H_6 pressure decay sorption curves at fugacity 0.6.

Supplementary Fig. 18. C_2H_4 and C_2H_6 sorption isotherm at 308.15K.

Supplementary Fig. 19. XPS C 1s peak deconvolution results in pristine CMS.

Supplementary Fig. 20. XPS C 1s peak deconvolution results in 50kGy CMS.

Supplementary Fig. 21. XPS C 1s peak deconvolution results in 100kGy CMS.

Supplementary Fig. 22. XPS C 1s peak deconvolution results in 250kGy CMS.

Supplementary Fig. 23. Diagram of Wicke-Kallenbach (mixed gas permeation test)

Supplementary Fig. 24. Separation performance of highly irradiated CMS membranes with different pyrolysis temperatures.

Supplementary Fig. 25. 87K Argon adsorption isotherm.

Supplementary Fig. 26. Structural property comparison from 87K Argon physisorption.

Supplementary Fig. 27. Hypothetical scheme for the fresh and aged electron-irradiated CMS microstructure.

Supplementary Table 1. Performance of polymeric membranes for C_2H_4 permeability and C_2H_4/C_2H_6 selectivity.

Supplementary Table 2. The carbon molecular sieve (CMS) membranes for C_2H_4 permeability and C_2H_4/C_2H_6 selectivity

Supplementary Table 3. The mixed matrix membranes (MMMs) for C_2H_4 permeability and C_2H_4/C_2H_6 selectivity

Supplementary Table 4. MOF membranes for C_2H_4 permeability and C_2H_4/C_2H_6 selectivity

Supplementary Table 5. The polymeric membranes for H_2 permeability and H_2/CO_2 selectivity

Supplementary Table 6. The carbon molecular sieve (CMS) membranes for H_2 permeability and H_2/CO_2 selectivity

Supplementary Table 7. The mixed matrix membranes (MMMs) for H_2 permeability and H_2/CO_2 selectivity

Supplementary Table 8. MOF membranes for H_2 permeability and H_2/CO_2 selectivity

Supplementary Table 9. Elemental fraction of pristine- and 100kGy-CMS films pyrolyzed at 500°C

Characterization of CMS membranes

Raman spectroscopy (Aramis, Horiba)

Raman spectra analysis was obtained from Aramis dispersive Raman spectrometer(Aramis, Horiba) using 514nm laser.

X-ray photoelectron spectroscopy (Axis-Supra, Kratos)

Survey scan and depth profiling for each sample was recorded with In-Situ X-ray photoelectron spectroscopy (Axis-Supra, Kratos). During depth profiling, gas cluster ion source was used as sputter ion, and the etching process was carried out every 10 seconds until the atomic composition becomes constant.

Surface profiler (Dektak-8, Veeco)

The etched depth of CMS surface from XPS ion sputter was scanned through surface profiler (Dektak-8, Veeco). The profiler with a diamond-tipped stylus scans across the sample surface and the recorded vertical motion of the stylus reveals the specific height information on the sample.

Transmission electron microscope (Titan cubed G2 60-300, FEI)

High-resolution transmission electron microscopy(HRTEM) was carried out on Titan Double Cs corrected TEM (Titan cubed G2 60-300, FEI) with acceleration voltage at 300kV. For specimen preparation, CMS film piece was placed in a mold and covered with epoxy resin to harden it. The prepared specimens were cut into thin slice by ultramicrotomy (Ultracut EM UC7, LEICA). The obtained TEM image was made binary in ImageJ for better visualizing the CMS structure.

Scanning electron microscope (Magellan400, FEI)

Scanning electron microscopy images were obtained at 10kV accelerating voltage and 0.64nA emission current. Before loading to SEM, the CMS film was attached to sample mount. Then the CMS film was coated with Au using sputter to increase the sample conductivity. With the SEM analysis, EDS line scan was also carried out to quantify oxygen composition sub 10μm CMS surface.

Argon physisorption (3Flex, Micrometrics)

87K Argon physisorption was carried out using 3flex(Micrometrics). Specific surface area and pore volume can be acquired by measuring the amount of argon that physically adsorb into the surface and pores of the porous sample according to the change of the relative pressure (P/P_0) at the liquid argon temperature (87K).

Elemental analysis (FlashEA 1112, Thermo Finnigan)

Elemental analysis of the fresh and aged CMS film (pristine and 100kGy) was carried out Flash 1112 (Thermo Finnigan) for C, H, N analysis, and oxygen fraction was estimated by Flash 2000 series (Thermo Scientific).

Supplementary Note 1. Fickian transport diffusion coefficient determination.

The Fickian transport diffusion coefficient(D) of ethylene(C_2H_4) and ethane(C_2H_6) in the CMS was determined using a sorption decay test. Kinetic sorption graphs show the amount of normalized target gas adsorbed on CMS over time, allowing diffusion coefficients to be calculated by fitting the curves with Fickian solutions.[1] First, using the target gas's compressibility at 35°C, the resultant pressure change can be expressed using normalized form **Equation S1**.

$$\frac{M_t}{M_\infty} \quad (1)$$

M_t is the amount of absorbed gas on CMS at time t and M_∞ is the corresponding total sorption amount after infinite time ∞.

The kinetic uptake curve (**Figure S10-12**) was fitted with the Fickian model using MATLAB. **Equation S2** is the applied infinite series solution for a thin film membrane with a half thickness l.

$$\frac{M_t}{M_\infty} = 1 - \frac{8}{\pi^2}\sum_{n=0}^{\infty}\frac{\exp\{-(2n+1)^2\pi^2 \cdot \frac{Dt}{4l^2}\}}{(2n+1)^2} \quad (2)$$

Supplementary Note 2. Estimation apparent activation energy of permeation using Arrhenius regression

The Arrhenius relationship between gas permeability and testing temperature was used to derive the apparent activation energies for the CMS. The product of diffusivity (D) and sorption coefficient (S) is gas permeability (P, Barrer).

$$P = D \times S \qquad (3)$$

The Arrhenius equation (**Equation S4**) and the Van't Hoff equation (**Equation S5**) can be used to define the diffusivity and solubility coefficients, respectively.[2]

$$D = D_0 e^{-\frac{E_D}{RT}} \qquad (4)$$

$$S = S_0 e^{-\frac{H_S}{RT}} \qquad (5)$$

D_0 and S_0 are the pre-exponential factor of diffusion and sorption. The apparent diffusion activation energy and apparent heat of sorption, respectively, are E_D and H_S. From the equation S3, permeability P can be written as following form,

$$P = P_0 e^{-\frac{E_P}{RT}} \qquad (6)$$

Where, $E_P = E_D \times H_S$ is the apparent activation energy. When **equation S6** is logarithmic on both sides, E_P can be determined by linear regression of the $\ln P$ versus $1/T$.

$$\ln P = \ln P_0 - \frac{E_P}{R}\frac{1}{T} \qquad (7)$$

Based on the 35 °C, 45 °C and 55 °C H_2 and CO_2 permeability data, P_0 and E_P can be calculated. From the completed equation, the predicted permeability value at a specific temperature can be obtained through linear regression.

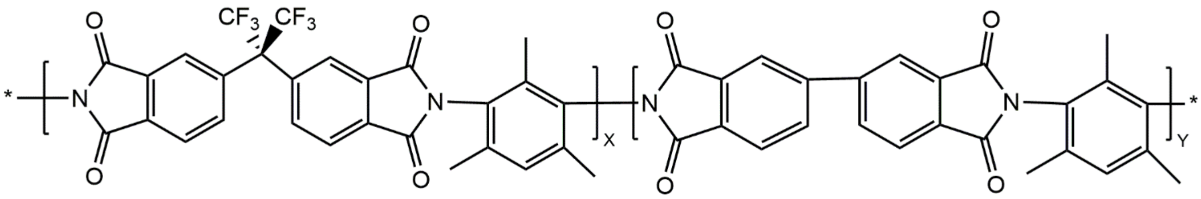

Supplementary Fig. 1. Chemical structure of polyimide 6FDA:BPDA(1:1)-DAM (X=1, Y=1).

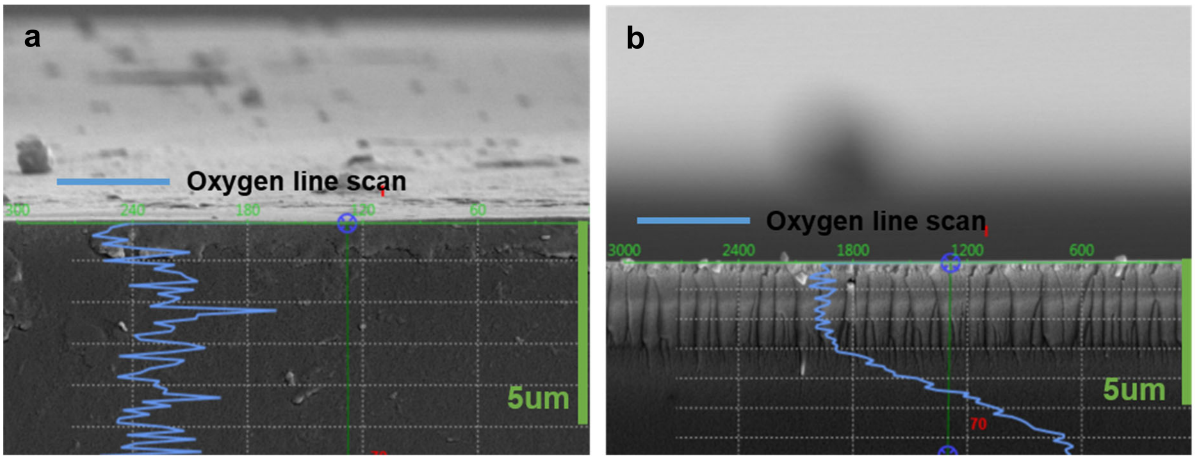

Supplementary Fig. 2. Oxygen line scan from Energy-dispersive X-ray spectrometer (EDS) (a) Oxygen versus CMS depth (10μm) of 500°C pristine-CMS and (b) 500kGy-CMS.

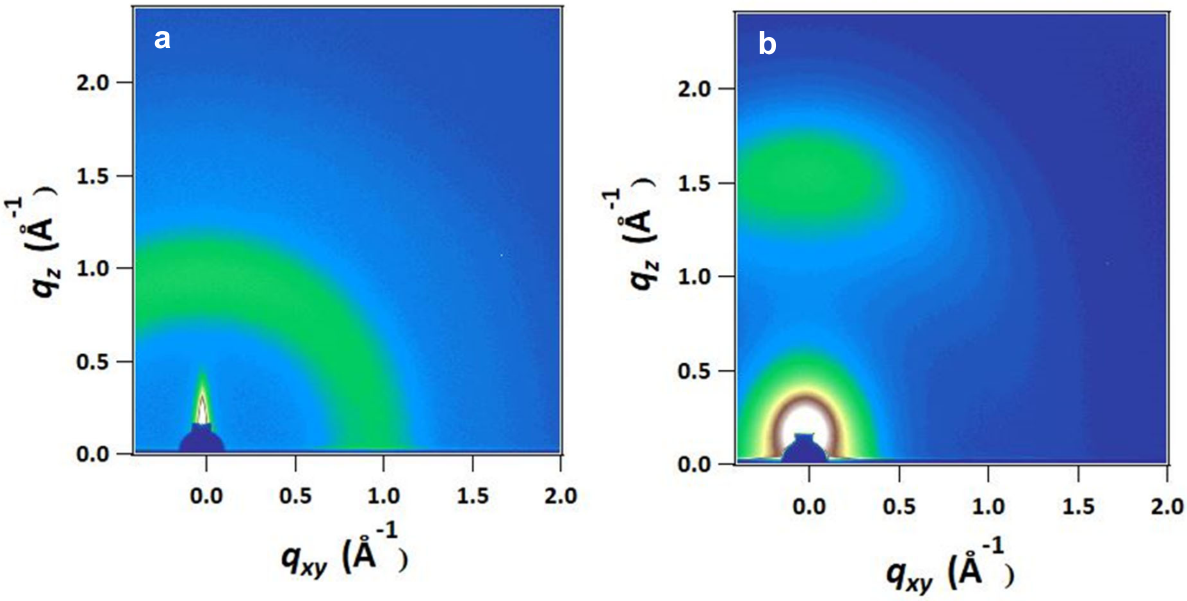

Supplementary Fig. 3. Grazing-incidence wide-angle X-ray scattering (GI-WAXS) 2D scattering image. (a) GIWAXS 2D scattering image of polymer precursor film and (b) 600°C pyrolyzed pristine CMS sample.

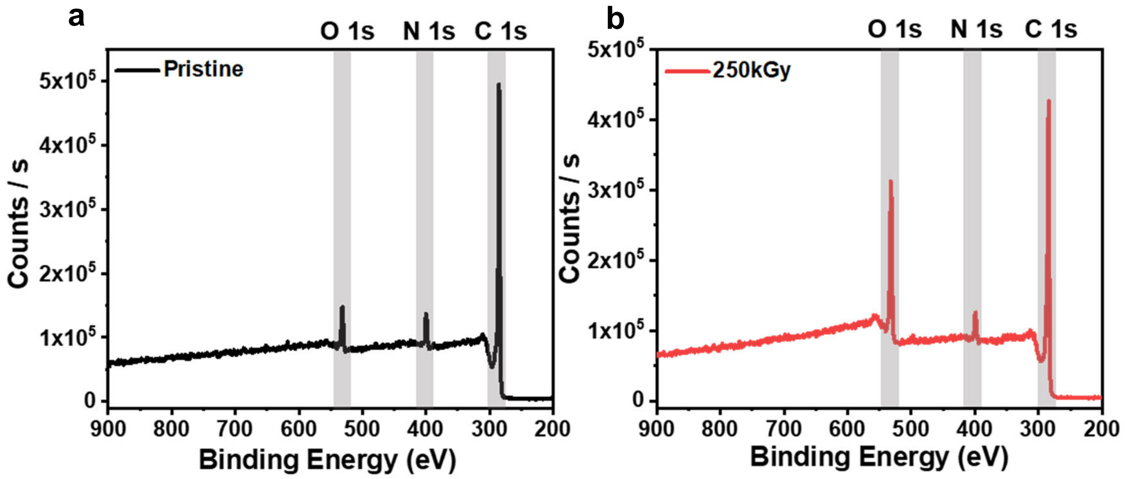

Supplementary Fig. 4. X-ray photoelectron spectroscopy (XPS) survey scan. (a) XPS survey scan of 500 °C pristine-CMS. (b) XPS survey scan of 250kGy-CMS without y-axis arbitrary unit (Counts/s vs Binding energy). The intensified O1s peak around 530eV become clear after electron irradiation. Source data are provided as a Source Data file.

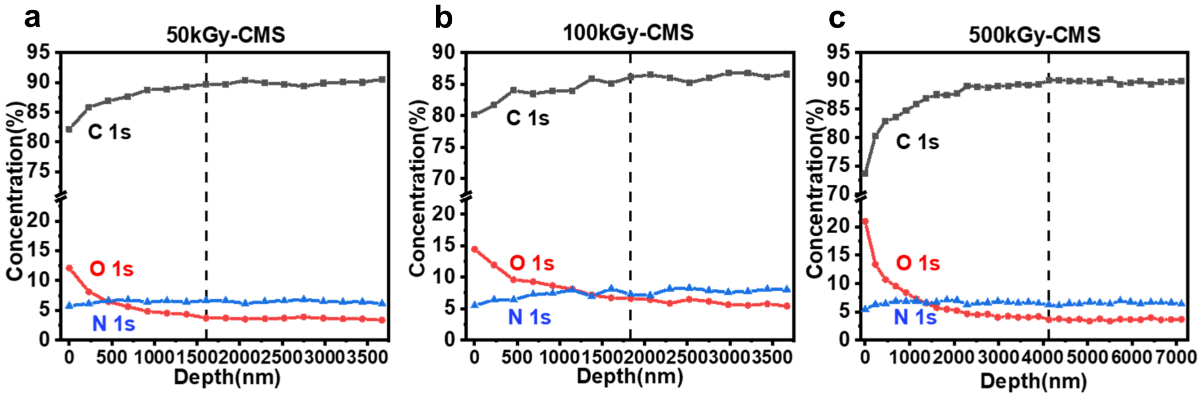

Supplementary Fig. 5. X-ray photoelectron spectroscopy (XPS) depth profiling. (a) depth profiling result of 500°C 50kGy-CMS, (b) 100kGy-CMS, and (c) 500kGy-CMS with respect to the C, N, O composition versus sample depth. Source data are provided as a Source Data file.

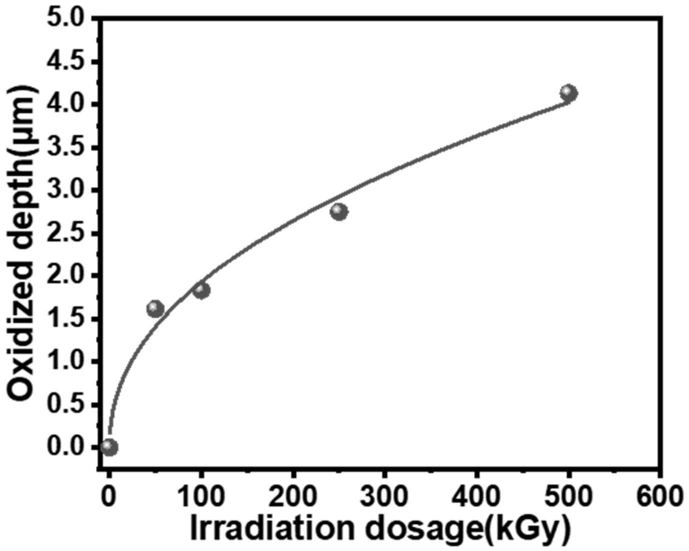

Supplementary Fig. 6. Oxidized carbon layer thickness as irradiation dosage. Thickness of oxidized carbon layer resulted from XPS depth profiling of 500°C pristine, 50kGy, 100kGy, 250kGy and 500kGy-CMS. Source data are provided as a Source Data file.

a

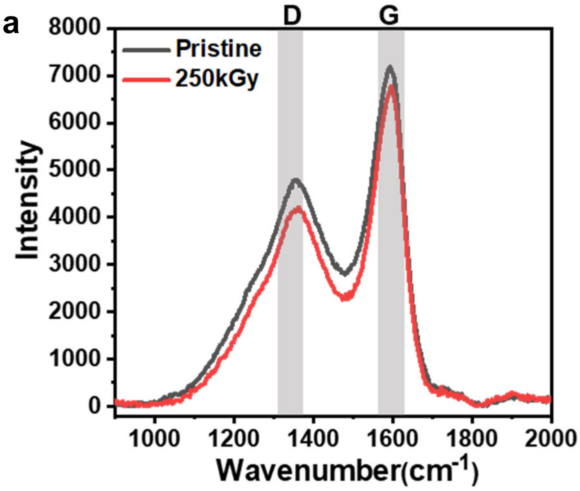

b

	Pristine-CMS	250kGy-CMS
I_D	4791.224	4214.635
I_G	7182.265	6778.74
I_D/I_G ratio	0.667	0.621

Supplementary Fig. 7. Results of Raman spectroscopy. (a) Raman spectrum of the 500 °C pristine- and 250kGy-CMS as a version without arbitrary unit. (b) Table of maximum value of D and G peak at each CMS, which gives the calculated I_D/I_G ratio. Source data are provided as a Source Data file.

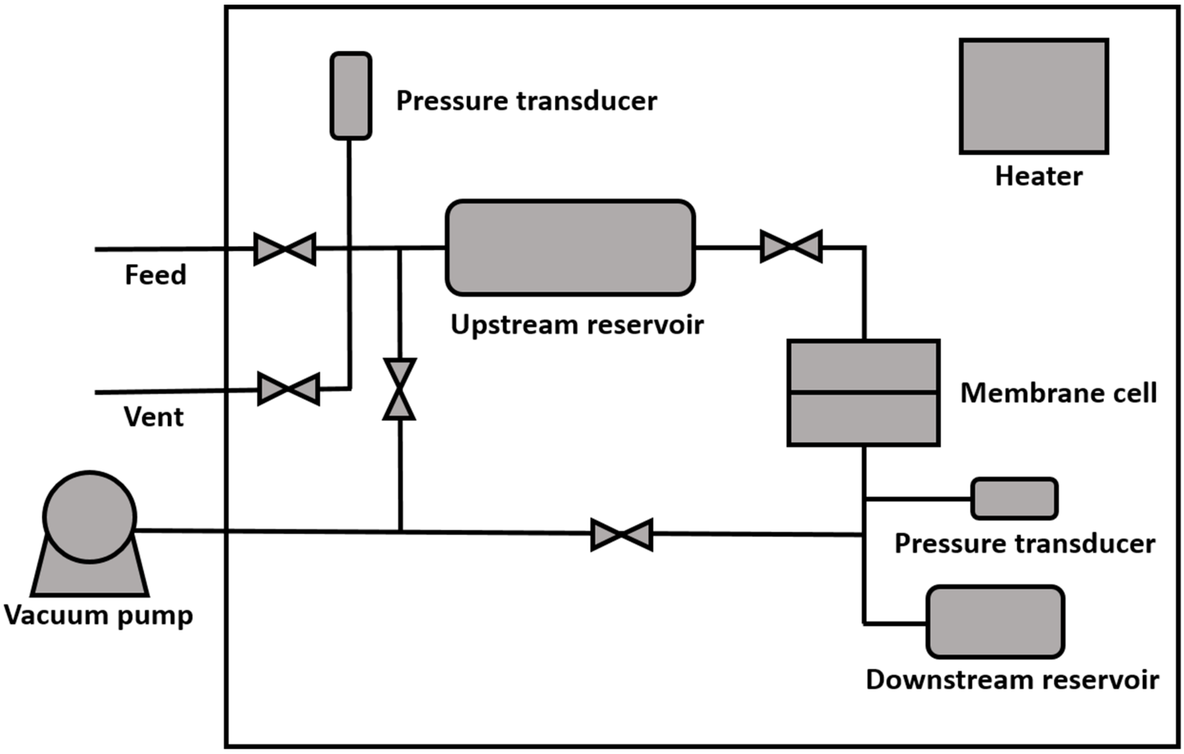

Supplementary Fig. 8. Schematic representation of constant-volume gas permeation apparatus.

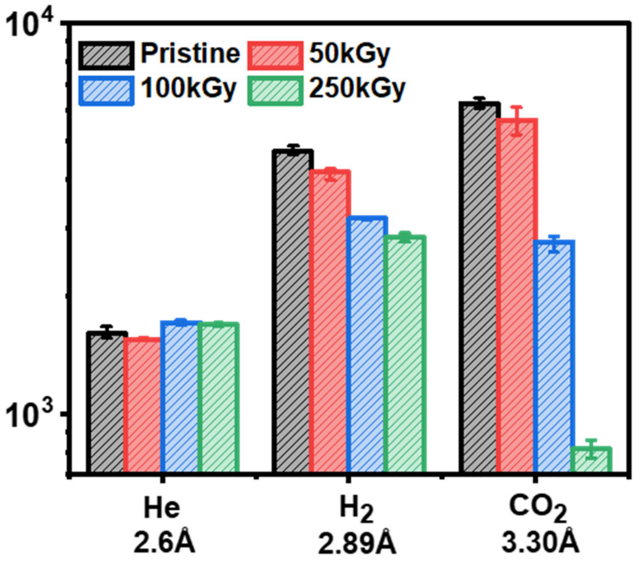

Supplementary Fig. 9. Separation performance of the prepared CMS membranes in He, H_2 and CO_2. Enlarged version of He, H_2 and CO_2 permeability with error bars (error bars indicate the standard deviation at three or four measurements) Source data are provided as a Source Data file.

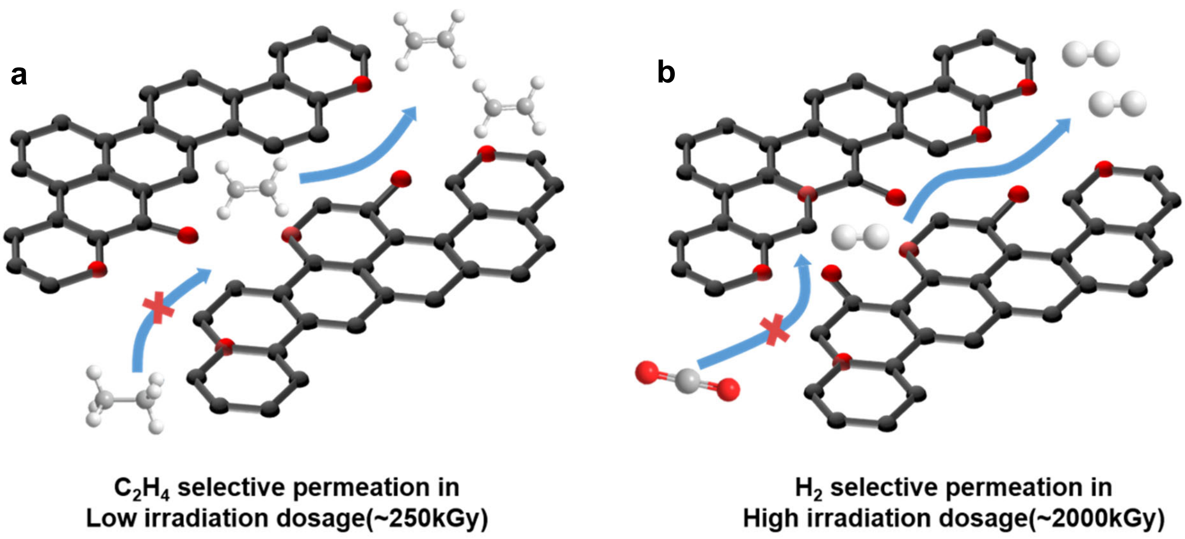

Supplementary Fig. 10. Schematic for selective gas permeation in CMS. (a) Selective C_2H_4 permeation with blocked C_2H_6 in low irradiation dosage. (b) Selective H_2/CO_2 permeation through highly irradiated CMS.

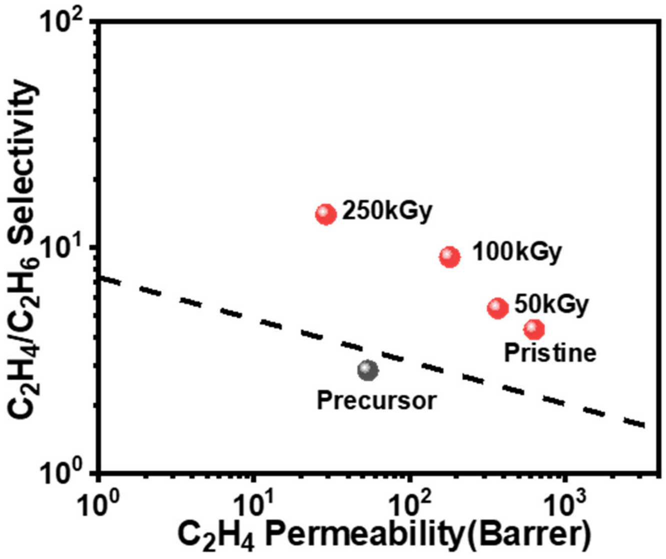

Supplementary Fig. 11. Separation performance of precursor and CMS membranes. C_2H_4 permeability vs C_2H_4/C_2H_6 selectivity data for 500 °C pristine- and electron-irradiated CMS membranes compared with polymeric precursor membrane performance. Source data are provided as a Source Data file.

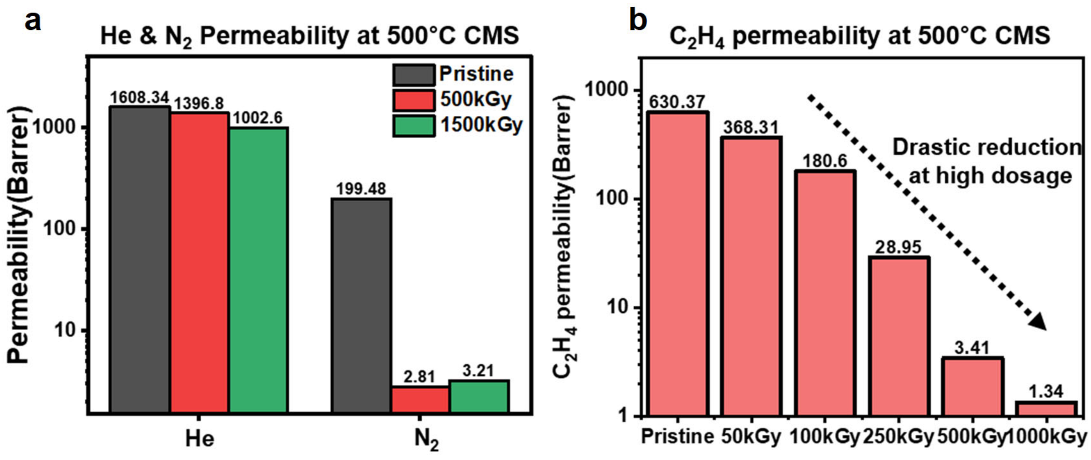

Supplementary Fig. 12. Gas permeation data of highly irradiated CMS membranes. (a) He and N_2 permeability of 500 °C pristine, 500kGy- and 1500kGy-CMS. (b) C_2H_4 permeation data of 500 °C pristine- and electron irradiated (up to 1000kGy) CMS membranes. Source data are provided as a Source Data file.

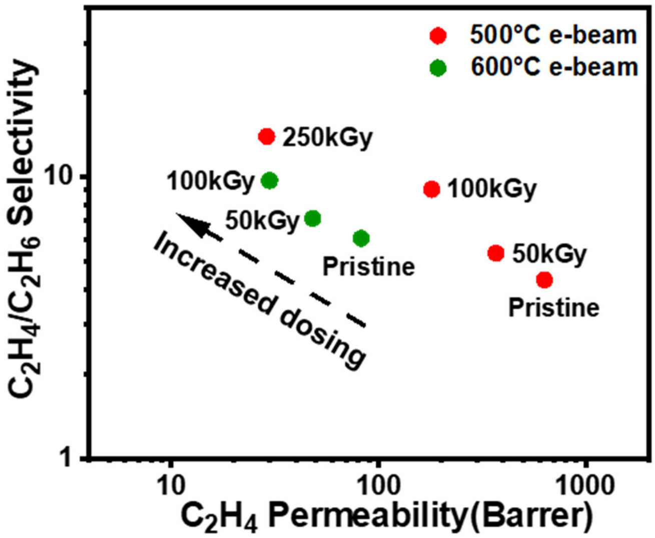

Supplementary Fig. 13. Permeation data of 600 °C CMS membranes including 500 °C data. Separation performance of electron irradiated 500 °C and 600 °C CMS membranes. Source data are provided as a Source Data file.

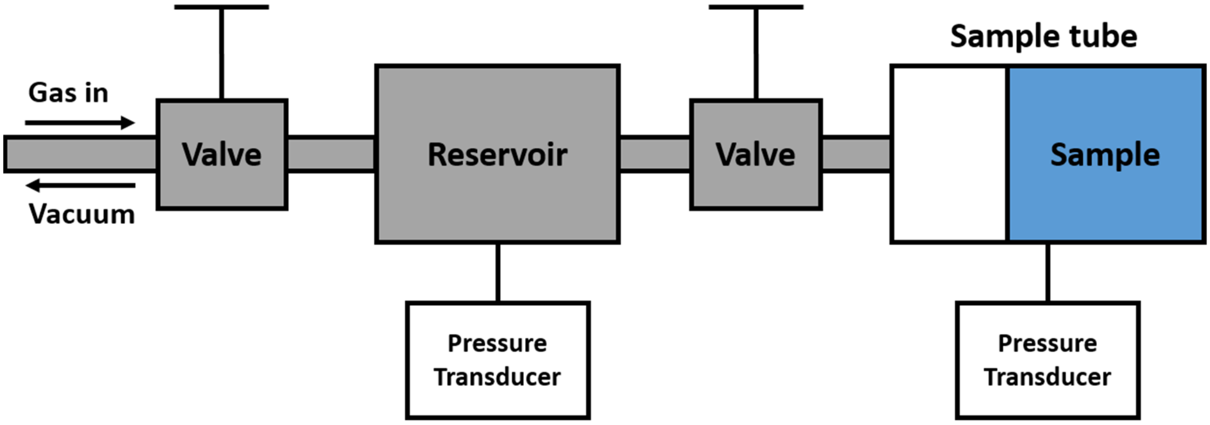

Supplementary Fig. 14. Custom-built pressure decay system illustration.

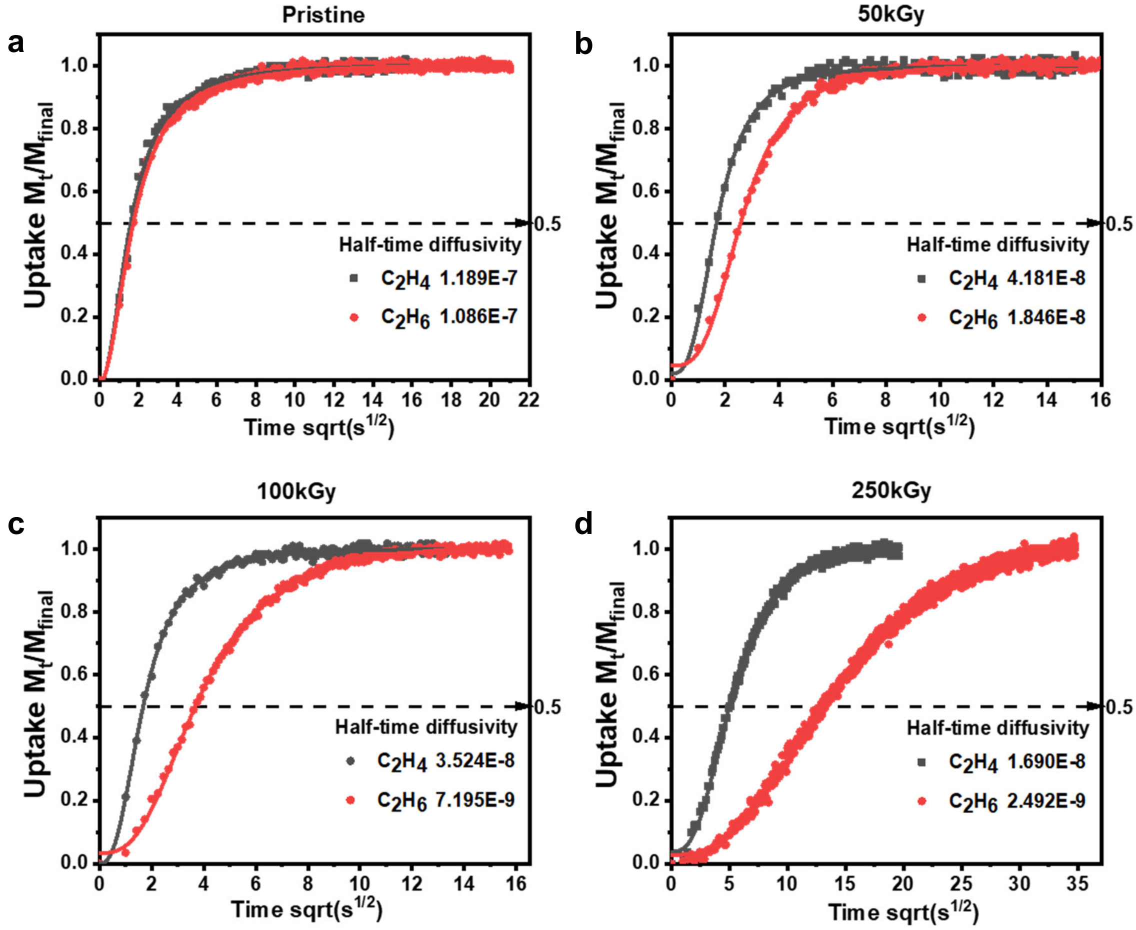

Supplementary Fig. 15. C_2H_4 and C_2H_6 pressure decay sorption curves at fugacity 0.1. (a) C_2H_4 and C_2H_6 sorption curve of 500°C pristine CMS films, (b) 50kGy CMS films, (c) 100kGy CMS films, and (d) 250kGy CMS films at fugacity 0.1. Source data are provided as a Source Data file.

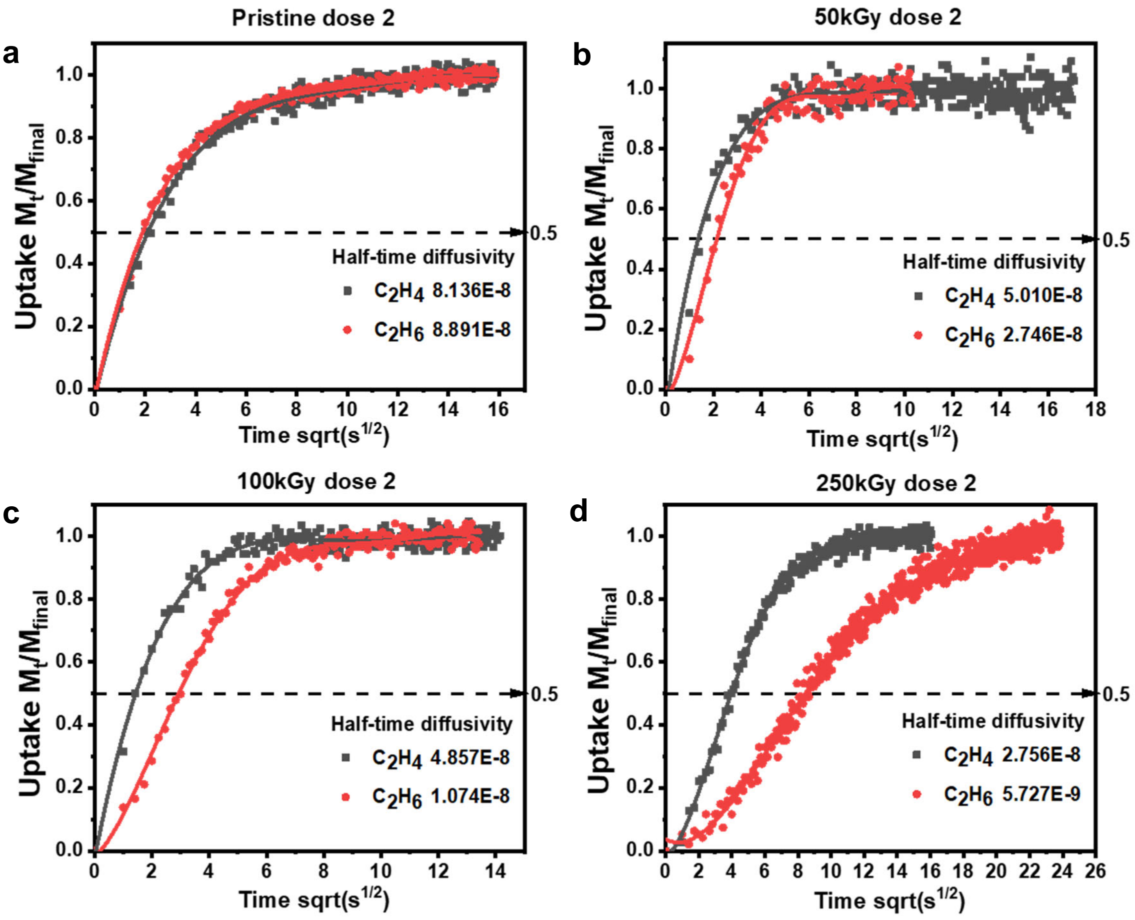

Supplementary Fig. 16. C_2H_4 and C_2H_6 pressure decay sorption curves at fugacity 0.3. (a) C_2H_4 and C_2H_6 sorption curve of 500°C pristine CMS films, (b) 50kGy CMS films, (c) 100kGy CMS films, and (d) 250kGy CMS films at fugacity 0.3. Source data are provided as a Source Data file.

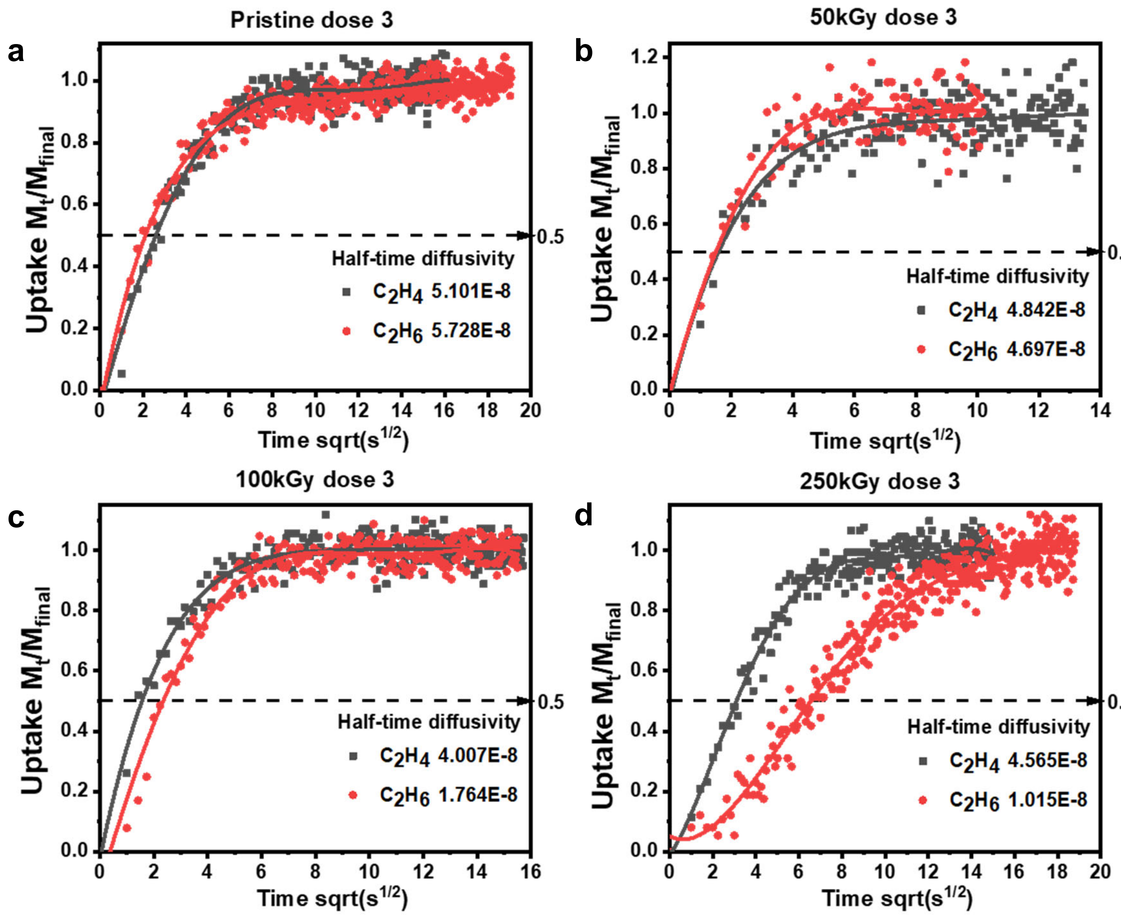

Supplementary Fig. 17. C_2H_4 and C_2H_6 pressure decay sorption curves at fugacity 0.6. (a) C_2H_4 and C_2H_6 sorption curve of 500°C pristine CMS films, (b) 50kGy CMS films, (c) 100kGy CMS films, and (d) 250kGy CMS films at fugacity 0.6. Source data are provided as a Source Data file.

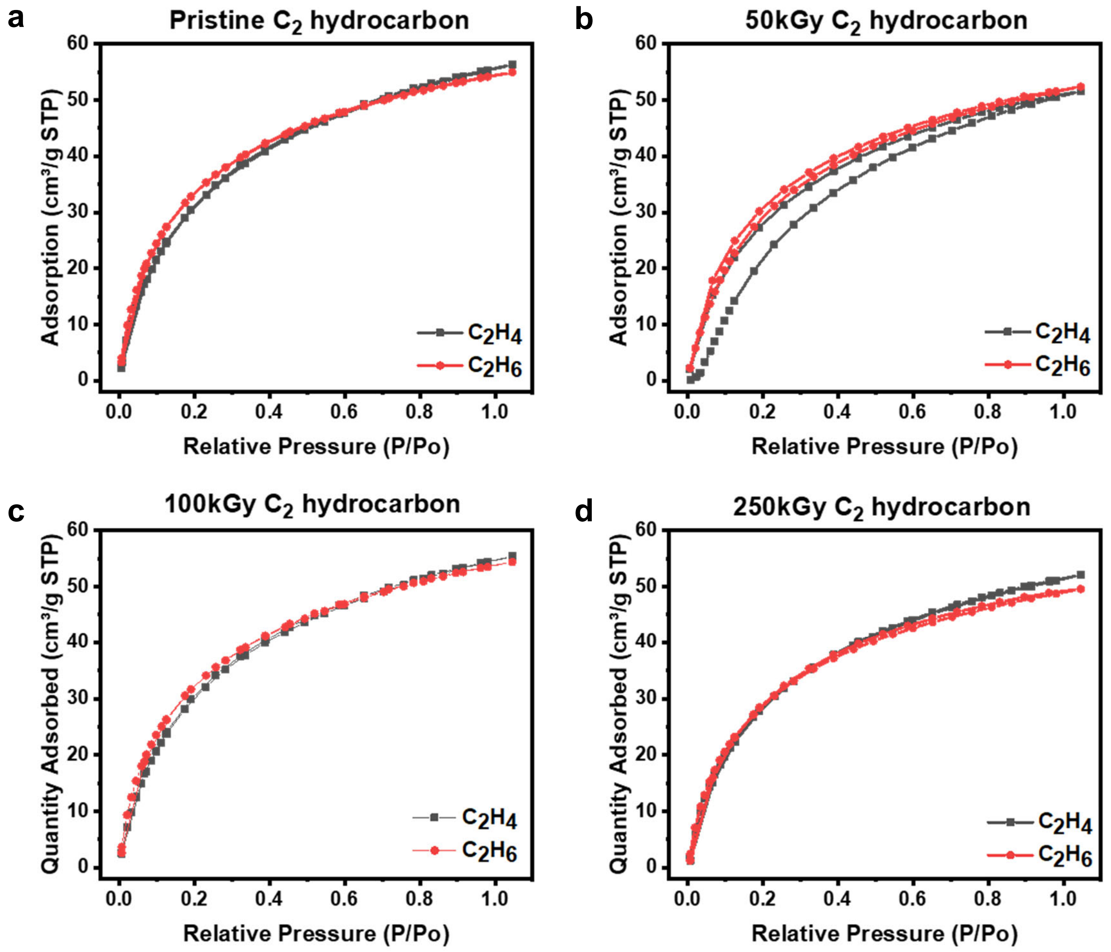

Supplementary Fig. 18. C_2H_4 and C_2H_6 sorption isotherm at 308.15K. (a) 500°C pristine-CMS, (b) 50kGy-CMS, (c) 100kGy-CMS and (d) 250kGy-CMS at 308.15K. Source data are provided as a Source Data file.

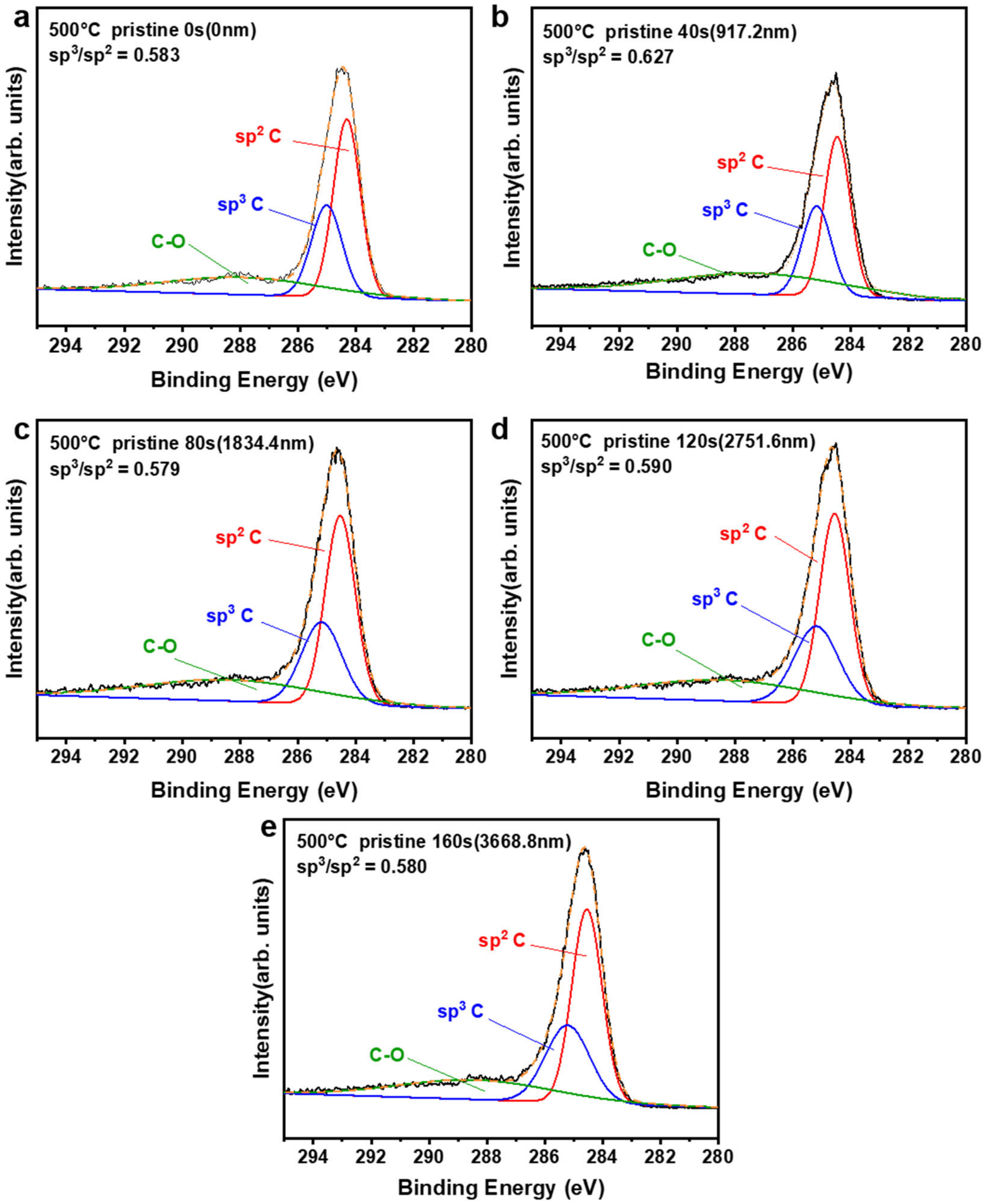

Supplementary Fig. 19. XPS C 1s peak deconvolution results in pristine CMS. (a) XPS sp^3/sp^2 ratio of 500 °C pristine CMS at (a) surface, (b) etched depth 917.2nm, (c) etched depth 1834.4nm (d) etched depth 2751.6nm and (e) etched depth 3668.8nm. Source data are provided as a Source Data file.

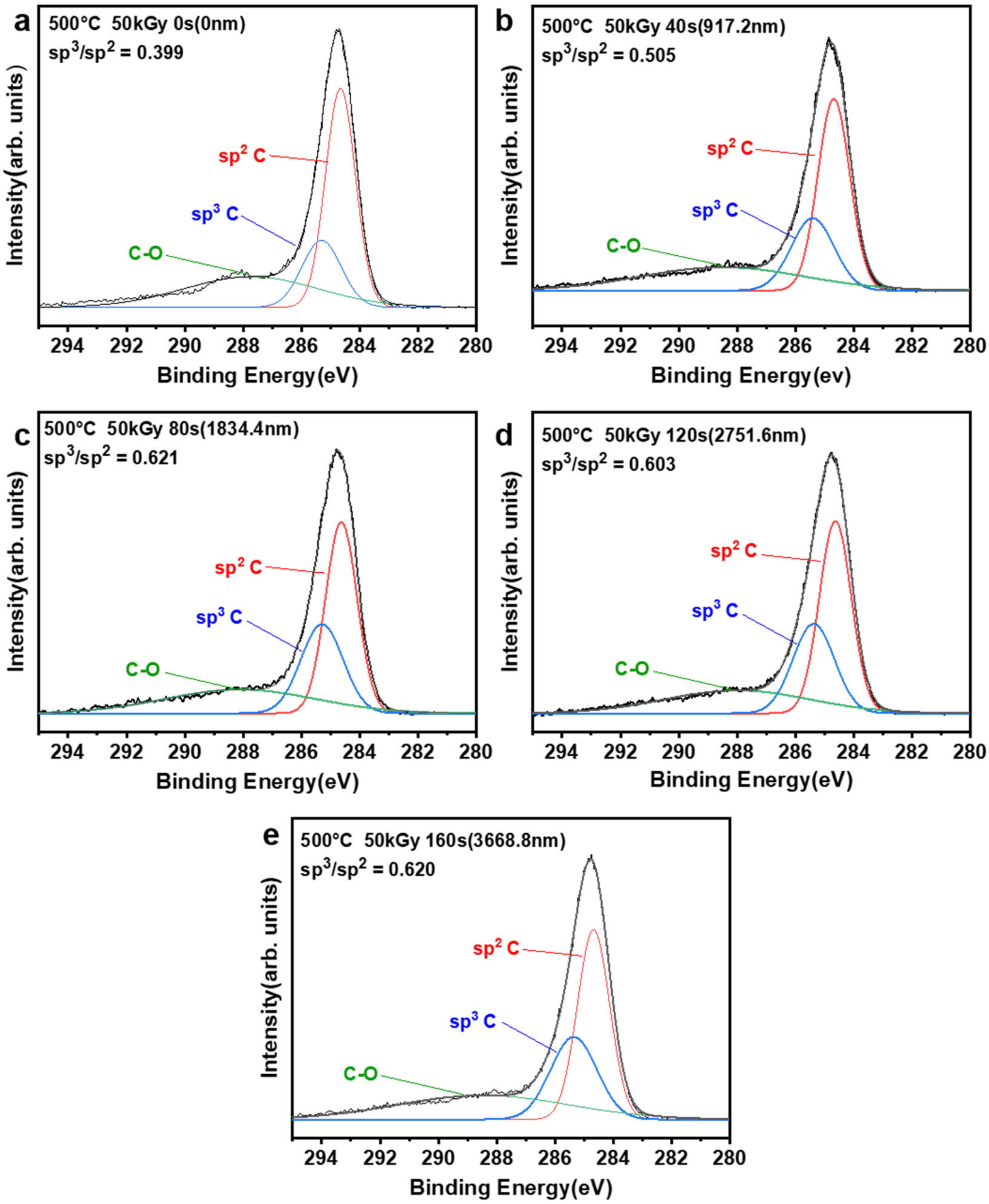

Supplementary Fig. 20. XPS C 1s peak deconvolution results in 50kGy CMS. (a) XPS sp^3/sp^2 ratio of 500 °C 50kGy CMS at (a) surface, (b) etched depth 917.2nm, (c) etched depth 1834.4nm (d) etched depth 2751.6nm and (e) etched depth 3668.8nm. Source data are provided as a Source Data file.

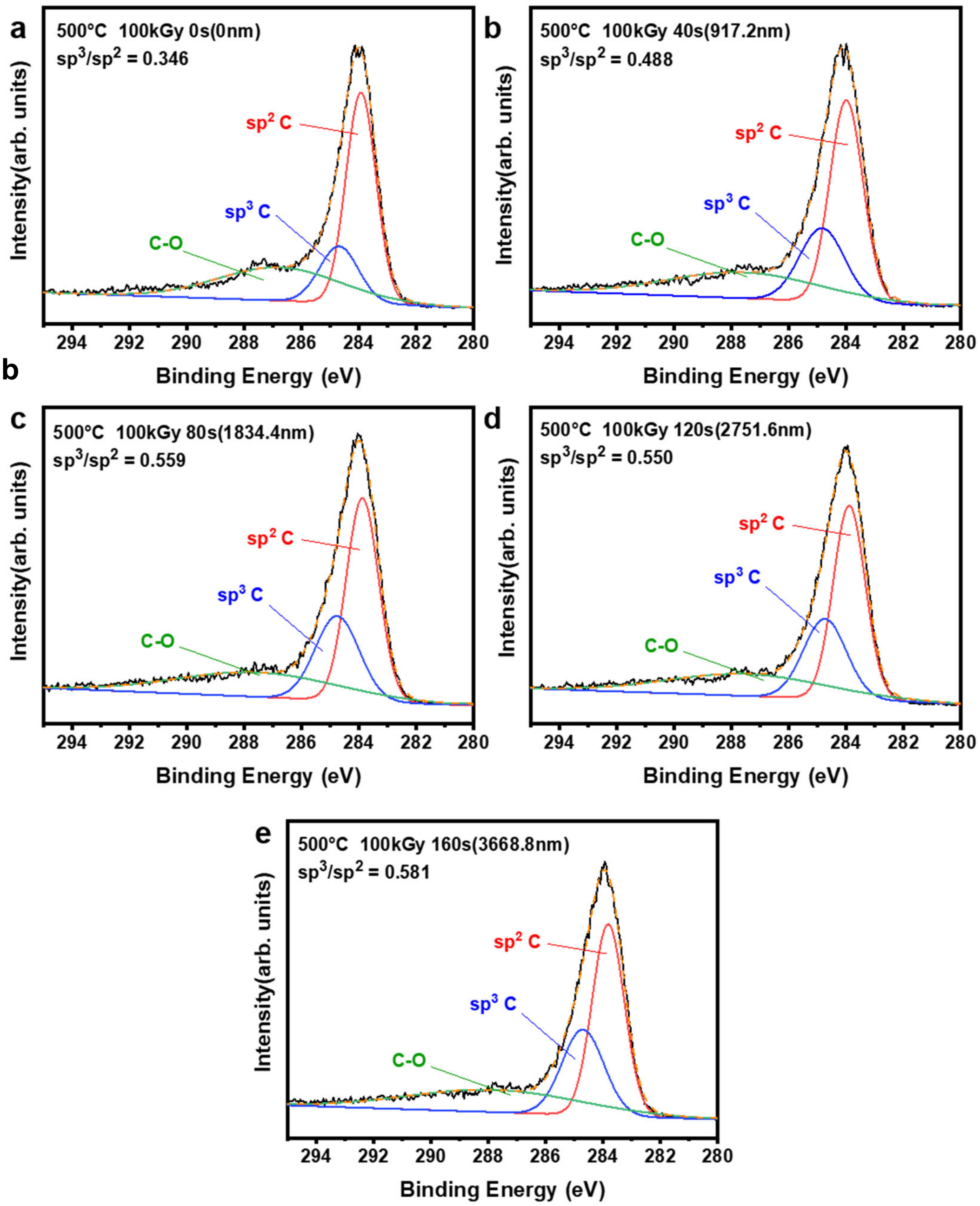

Supplementary Fig. 21. XPS C 1s peak deconvolution results in 100kGy CMS. (a) XPS sp^3/sp^2 ratio of 500 °C 100kGy CMS at (a) surface, (b) etched depth 917.2nm, (c) etched depth 1834.4nm (d) etched depth 2751.6nm and (e) etched depth 3668.8nm. Source data are provided as a Source Data file.

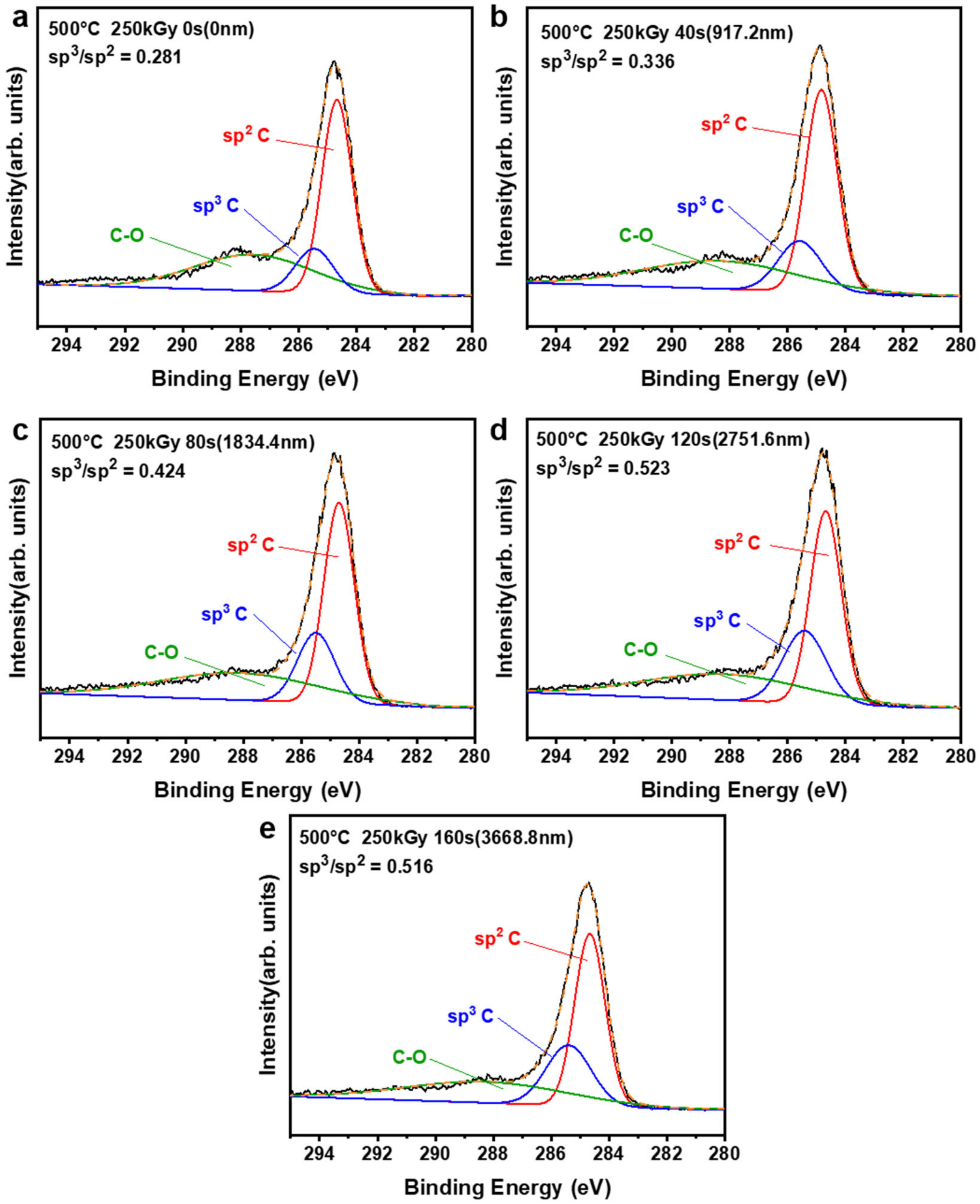

Supplementary Fig. 22. XPS C 1s peak deconvolution results in 250kGy CMS. (a) XPS sp^3/sp^2 ratio of 500 °C 250kGy CMS at (a) surface, (b) etched depth 917.2nm, (c) etched depth 1834.4nm (d) etched depth 2751.6nm and (e) etched depth 3668.8nm. Source data are provided as a Source Data file.

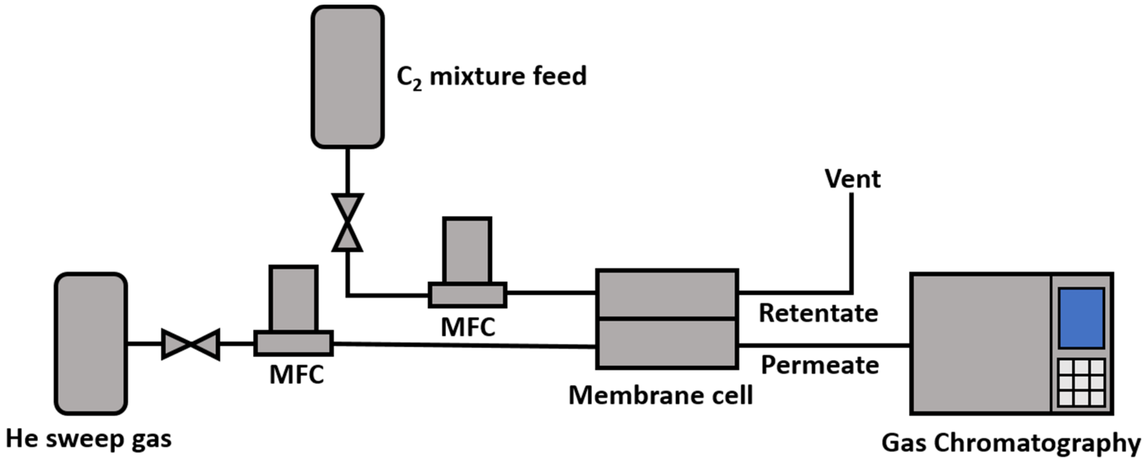

Supplementary Fig. 23. Diagram of Wicke-Kallenbach (mixed gas permeation test) system. Mixed gas permeation system for measuring separation performance of the prepared CMS membranes.

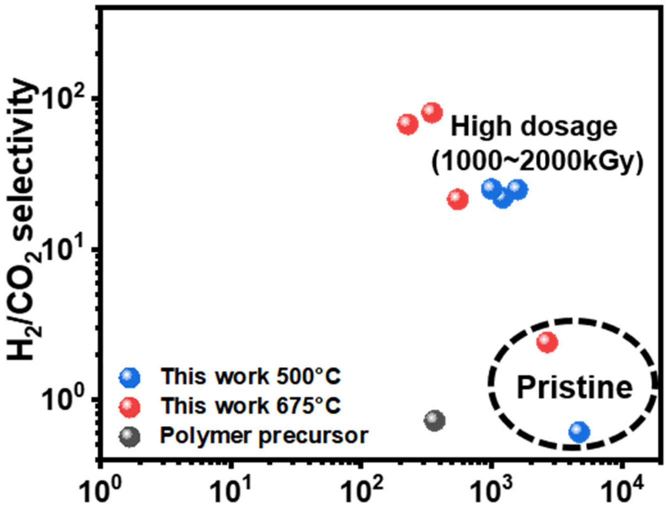

Supplementary Fig. 24. Separation performance of highly irradiated CMS membranes with different pyrolysis temperatures. H_2/CO_2 permeation data of pristine and highly electron-irradiated CMS membranes for two different temperatures (500 °C and 675 °C) including polymeric precursor membrane. Source data are provided as a Source Data file.

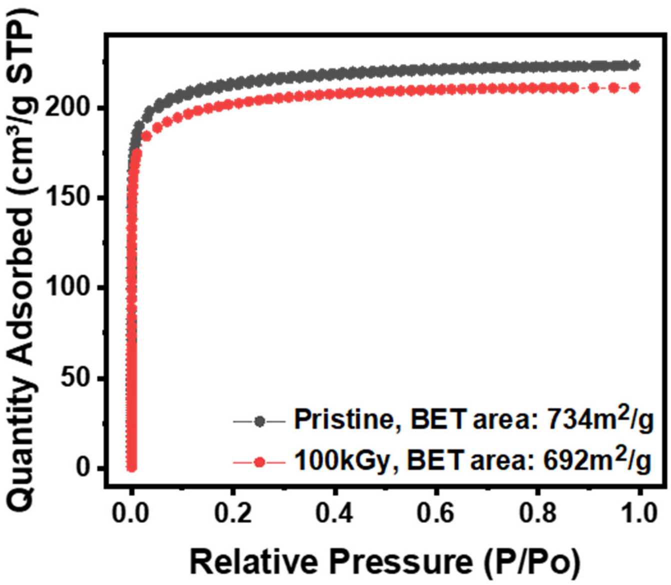

Supplementary Fig. 25. 87K Argon adsorption isotherm. 87k Argon physisorption data for 500 °C pristine- and 100kGy-CMS. Source data are provided as a Source Data file.

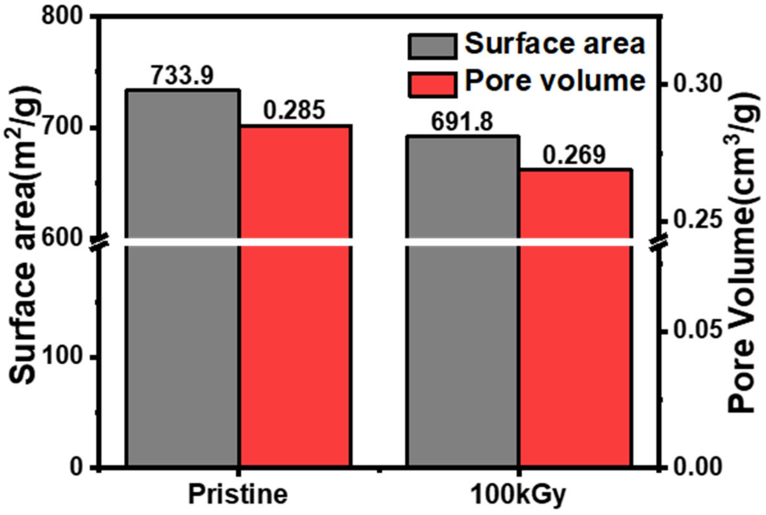

Supplementary Fig. 26. Structural property comparison from 87K Argon physisorption. BET surface area and pore volume of 500 °C pristine- and 100kGy-CMS. Source data are provided as a Source Data file.

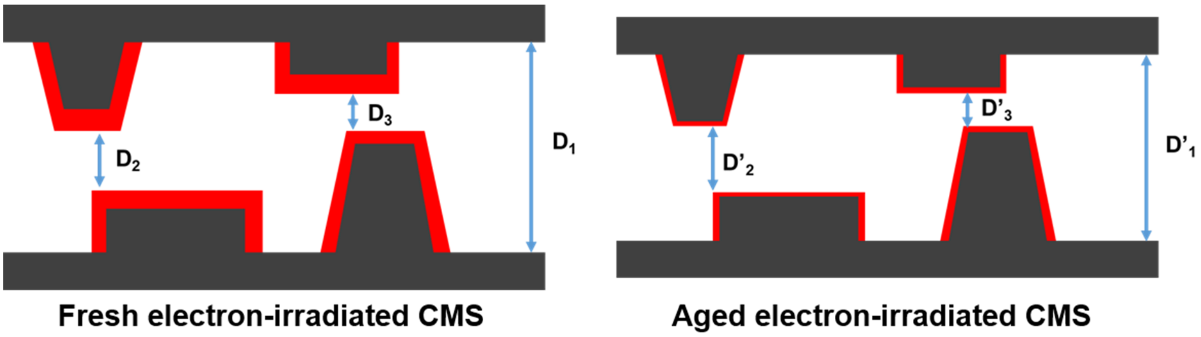

Supplementary Fig. 27. Hypothetical scheme for the fresh and aged electron-irradiated CMS microstructure. The slit-like structure of CMS composed of supermicropore (D_1 and D'_1) and ultramicropore (D_2, D'_2, D_3 and D'_3).

Supplementary Table 1. Performance of polymeric membranes for C_2H_4 permeability and C_2H_4/C_2H_6 selectivity.

**Materials**	**C_2H_4(Barrer)**	**Selectivity**	**Test condition**
Matrimid®[3]	0.45	4.5	35°C, 3.4atm(50psia), single gas
6FDA-DAM[3]	64	3	35°C, 3.4atm(50psia), single gas
6FDA:BPDA-DAM[3]	46	3.3	35°C, 3.4atm(50psia), single gas
6FDA-Durene[4]	76.7	2.9	35°C, 2atm(29.4psia), single gas
6FDA-NDA[4]	1.17	6.8	35°C, 2atm(29.4psia), single gas
6FDA-NDA/Durene(75:25)[4]	4.46	5.6	35°C, 2atm(29.4psia), single gas
6FDA-NDA/Durene(50:50)[4]	9.48	4.3	35°C, 2atm(29.4psia), single gas
6FDA-NDA/Durene(25:75)[4]	36.7	3.6	35°C, 2atm(29.4psia), single gas
6FDA-mPD[5]	0.3	3.3	35°C, 3.8atm(55.8psia), single gas
6FDA-IPDA[5]	1.4	3.8	35°C, 3.8atm(55.8psia), single gas
6FDA-6FpDA[5]	2.1	4.4	35°C, 3.8atm(55.8psia), single gas
BPDA-TeMPD[6]	5.8	4.3	50°C, 2atm(29.4psia), single gas
6FDA-TrMPD[6]	58	2.9	50°C, 2atm(29.4psia), single gas

Supplementary Table 2. The carbon molecular sieve (CMS) membranes for C_2H_4 permeability and C_2H_4/C_2H_6 selectivity

Materials	C_2H_4(Barrer)	Selectivity	Test condition
Matrimid®[7]	14.5	6.6	35°C, 3.4atm(50psia), single gas
Matrimid®[7]	16.7	11.7	35°C, 3.4atm(50psia), single gas
PIM-1[8]	44	6.3	35°C, 2bar(29psia), single gas
PIM-1[8]	1.3	13	35°C, 2bar(29psia), single gas
PIM-6FDA-OH[9]	276	2.9	35°C, 2bar(29psia), single gas
PIM-6FDA-OH[9]	66.1	6.5	35°C, 2bar(29psia), single gas
PIM-6FDA-OH[9]	10.4	17.5	35°C, 2bar(29psia), single gas
PIM-6FDA[10]	328	2.1	35°C, 2atm(29.4psia), single gas
PIM-6FDA[10]	77	4.1	35°C, 2atm(29.4psia), single gas
PIM-6FDA[10]	3	25	35°C, 2atm(29.4psia), single gas
6FDA:BPDA-DAM[3]	135	4.8	35°C, 3.4atm(50psia), single gas
6FDA:BPDA-DAM[3]	78	6.4	35°C, 3.4atm(50psia), single gas
6FDA:BPDA-DAM[3]	53	7.8	35°C, 3.4atm(50psia), single gas
6FDA:BPDA-DAM[3]	13	9.2	35°C, 3.4atm(50psia), single gas
6FDA-DAM[11]	38	3.3	35°C, 3.4atm(50psia), single
6FDA-DAM[11]	28	7	35°C, 3.4atm(50psia), single
6FDA-DAM[11]	10	7.5	35°C, 3.4atm(50psia), single
BPDA-pp'ODA[12]	43	6.9	35°C, 1atm(14.7psia)
novolac-type phenolic resin[13]	110	4.9	20°C, 1bar, single gas
novolac-type phenolic resin[13]	860	4.3	20°C, 1bar, single gas
Fe-6FDA-DAM:DABA(3:2)[14]	60	9	35°C, 3.4atm(50psia), single gas
phenolic resin[15]	30	5.3	20°C, single gas
phenolic resin[15]	370	3	20°C, single gas
Kapton polyimide[16]	55	5.5	100°C, single gas
Pristine 6FDA:BPDA(1:1)-DAM	630.4	4.32	35°C, 2bar(29.4psia), single gas
50kGy 6FDA:BPDA(1:1)-DAM	368.3	5.37	35°C, 2bar(29.4psia), single gas
100kGy 6FDA:BPDA(1:1)-DAM	180.6	9.06	35°C, 2bar(29.4psia), single gas
250kGy 6FDA:BPDA(1:1)-DAM	28.95	13.96	35°C, 2bar(29.4psia), single gas

Supplementary Table 3. The mixed matrix membranes (MMMs) for C_2H_4 permeability and C_2H_4/C_2H_6 selectivity

Materials	C_2H_4(Barrer)	Selectivity	Test condition
6FDA-DAM/Co2(dobdc) 33wt%[17]	180	5	35 °C, 2bar, single gas
6FDA-DAM/Ni2(dobdc) 25wt%[17]	330	4.7	35 °C, 2bar, single gas
6FDA-DAM/Mg2(dobdc) 23wt%[17]	1020	2.7	35 °C, 2bar, single gas
6FDA-DAM/Mn2(dobdc) 13wt%[17]	450	2.3	35 °C, 2bar, single gas
6FDA-DAM/Ni2(dobdc) 6wt%[17]	210	4	35 °C, 2bar, single gas
6FDA-DAM/Co2(dobdc) 10wt%[17]	150	3.8	35 °C, 2bar, single gas
ZIF-8@DBzPBI-BuI[18]	110	2.6	35°C, 40psi(2.72bar), single gas
HKUST-1@6FDA-TMPDA[19]	183	2.4	35 °C, 2bar, mixed gas
Cu3BTC2@P84[20]	0.05	7.1	5bar, single gas
MOF-801@Ni-MOF-74 MMM(6FDA-durene)[21]	26.15	5.9	35 °C, 2bar, single gas
MOF-801@Ni-MOF-74 MMM(6FDA-DAM) [21]	33.2	5.3	35 °C, 2bar, single gas
Ni-gallate@6FDA-DAM[22]	91.9	3.5	25 °C, 1.5bar, single gas
Ni-gallate@6FDA-DAM[22]	74.8	2.5	25 °C, 1.5bar, mixed gas(1:1)
ZIF-8@PPEES[23]	3.15	2	30°C, 1bar, single gas

Supplementary Table 4. MOF membranes for C_2H_4 permeability and C_2H_4/C_2H_6 selectivity

**Materials**	**C_2H_4(Barrer)**	**Selectivity**	**Test condition**
ZIF-8[24]	1238	4.2	20 °C, 5bar, single gas
ZIF-8[25]	1074	2.3	25 °C, 1bar, single gas
ZIF-8[25]	1164	2	25 °C, 1bar, mixed gas(1:1)
ZIF-8[26]	237	2.1	25 °C, 1.1bar, mixed gas(1:1)

Supplementary Table 5. The polymeric membranes for H_2 permeability and H_2/CO_2 selectivity

**Materials**	**H_2(Barrer)**	**Selectivity**	**Test condition**
6FDA-durene crosslinked with TAEA[27]	300	47	35°C, 3.5 atm, single gas
6FDA-durene crosslinked with EDA[28]	52	130	35°C, 3.5 atm, single gas
Matrimid ®[29]	28	3.5	25°C, mixed-gas(1:1), 0.2 bar

Supplementary Table 6. The carbon molecular sieve (CMS) membranes for H_2 permeability and H_2/CO_2 selectivity

**Materials**	**H_2(Barrer)**	**Selectivity**	**Test condition**
Cellophane[30]	39	59	30℃, 2 bar, single gas
Kapton®[31]	7.2	161	50℃, 2 bar, single gas
PBI[32]	640	17	100℃, 7.4 atm, single gas
PBI[32]	2400	8.7	100℃, 7.4 atm, single gas
Matrimid®[33]	1200	4.5	35℃, 100psia, single gas
Matrimid®[33]	250	9	35℃, 100psia, single gas
PABZ-6FDA-PI[34]	460	8.1	35℃, 1bar, single gas
PPO[35]	1355	6.2	25℃, 1atm, single gas
Cellulose[36]	295.3	9.2	25℃, 1atm, single gas
Cellulose[36]	101	27.7	25℃, 1atm, single gas
Cellulose[36]	46.2	45.4	25℃, 1atm, single gas
Pristine 6FDA:BPDA(1:1)-DAM	4707.4	0.61	35℃, 2bar, single gas, 500 ℃
1000kGy 6FDA:BPDA(1:1)-DAM	1580.2	24.82	35℃, 2bar, single gas, 500 ℃
1500kGy 6FDA:BPDA(1:1)-DAM	1216.1	21.9	35℃, 2bar, single gas, 500 ℃
2000kGy 6FDA:BPDA(1:1)-DAM	1000.4	24.9	35℃, 2bar, single gas, 500 ℃
Pristine 6FDA:BPDA(1:1)-DAM	2670.2	2.4	35℃, 2bar, single gas, 675 ℃
1000kGy 6FDA:BPDA(1:1)-DAM	551.5	21.34	35℃, 2bar, single gas, 675 ℃
1500kGy 6FDA:BPDA(1:1)-DAM	349.4	80.45	35℃, 2bar, single gas, 675 ℃
2000kGy 6FDA:BPDA(1:1)-DAM	227.71	67.4	35℃, 2bar, single gas, 675 ℃

Supplementary Table 7. The mixed matrix membranes (MMMs) for H_2 permeability and H_2/CO_2 selectivity

**Materials**	**H_2(Barrer)**	**Selectivity**	**Test condition**
ZIF-71@6FDA-durene (Crosslinked with TAEA)[37]	270	50	35℃, 7atm, mixed-gas(1:1)
ZIF-7@PBI[38]	26	15	35℃, 3.5atm, single gas
ZIF-8@PBI[39]	105.4	12.3	35℃, 3.5atm, single gas

Supplementary Table 8. MOF membranes for H_2 permeability and H_2/CO_2 selectivity

Materials	H_2(Barrer)	Selectivity	Test condition
ZIF-8 membrane[40]	3582	6	25°C, 2bar, mixed gas(1:1)
ZIF-8 membrane[41]	6300	4.5	25°C, 1bar, single gas

Supplementary Table 9. Elemental fraction of pristine- and 100kGy-CMS films pyrolyzed at 500°C

	Weight fraction(%)			
	C	H	N	O
Fresh pristine-CMS	78.39	2.76	7.04	6.68
Aged (14 days) pristine-CMS	77.15	2.72	6.86	6.92
Fresh 100kGy-CMS	78.62	2.82	6.93	9.95
Aged (30 days) 100kGy-CMS	77.89	2.93	6.83	9.06

References

1. Crank, J. & Crank, E. P. J. *The Mathematics of Diffusion*. (Clarendon Press, 1979).

2. Rungta, M. *et al.* Carbon molecular sieve structure development and membrane performance relationships. *Carbon N. Y.* **115**, 237–248 (2017).

3. Rungta, M., Zhang, C., Koros, W. J. & Xu, L. Membrane-based ethylene/ethane separation: The upper bound and beyond. *AIChE J.* **59**, 3475–3489 (2013).

4. Chan, S. S., Chung, T.-S., Liu, Y. & Wang, R. Gas and hydrocarbon (C2 and C3) transport properties of co-polyimides synthesized from 6FDA and 1,5-NDA (naphthalene)/Durene diamines. *J. Memb. Sci.* **218**, 235–245 (2003).

5. Staudt-Bickel, C. & Koros, W. J. Olefin/paraffin gas separations with 6FDA-based polyimide membranes. *J. Memb. Sci.* **170**, 205–214 (2000).

6. Tanaka, K., Taguchi, A., Hao, J., Kita, H. & Okamoto, K. Permeation and separation properties of polyimide membranes to olefins and paraffins. *J. Memb. Sci.* **121**, 197–207 (1996).

7. Rungta, M., Xu, L. & Koros, W. J. Carbon molecular sieve dense film membranes derived from Matrimid® for ethylene/ethane separation. *Carbon N. Y.* **50**, 1488–1502 (2012).

8. Salinas, O., Ma, X., Litwiller, E. & Pinnau, I. Ethylene/ethane permeation, diffusion and gas sorption properties of carbon molecular sieve membranes derived from the prototype ladder polymer of intrinsic microporosity (PIM-1). *J. Memb. Sci.* **504**, 133–140 (2016).

9. Salinas, O., Ma, X., Litwiller, E. & Pinnau, I. High-performance carbon molecular sieve membranes for ethylene/ethane separation derived from an intrinsically microporous polyimide. *J. Memb. Sci.* **500**, 115–123 (2016).

10. Salinas, O., Ma, X., Wang, Y., Han, Y. & Pinnau, I. Carbon molecular sieve membrane from a microporous spirobisindane-based polyimide precursor with enhanced

ethylene/ethane mixed-gas selectivity. *RSC Adv.* **7**, 3265–3272 (2017).

11. Rungta, M. Carbon molecular sieve dense film membranes for ethylene/ethane separations. (Georgia Institute of Technology, 2012).

12. Hayashi, J.-I. *et al.* Separation of ethane/ethylene and propane/propylene systems with a carbonized BPDA- pp ‘ODA polyimide membrane. *Ind. Eng. Chem. Res.* **35**, 4176–4181 (1996).

13. Fuertes, A. B. & Menendez, I. Separation of hydrocarbon gas mixtures using phenolic resin-based carbon membranes. *Sep. Purif. Technol.* **28**, 29–41 (2002).

14. Chu, Y.-H. *et al.* Iron-containing carbon molecular sieve membranes for advanced olefin/paraffin separations. *J. Memb. Sci.* **548**, 609–620 (2018).

15. Centeno, T. A., Vilas, J. L. & Fuertes, A. B. Effects of phenolic resin pyrolysis conditions on carbon membrane performance for gas separation. *J. Memb. Sci.* **228**, 45–54 (2004).

16. Suda, H. & Haraya, K. Alkene/alkane permselectivities of a carbon molecular sieve membrane. *Chem. Commun.* 93–94 (1997).

17. Bachman, J. E., Smith, Z. P., Li, T., Xu, T. & Long, J. R. Enhanced ethylene separation and plasticization resistance in polymer membranes incorporating metal-organic framework nanocrystals. *Nat. Mater.* **15**, 845–849 (2016).

18. Kunjattu, S. H. *et al.* ZIF-8@DBzPBI-BuI composite membranes for olefin/paraffin separation. *J. Memb. Sci.* **549**, 38–45 (2018).

19. Chuah, C. Y., Samarasinghe, S. A. S. C., Li, W., Goh, K. & Bae, T.-H. Leveraging Nanocrystal HKUST-1 in Mixed-Matrix Membranes for Ethylene/Ethane Separation. *Membranes* **10**, (2020).

20. Ploegmakers, J., Japip, S. & Nijmeijer, K. Mixed matrix membranes containing MOFs for ethylene/ethane separation—Part B: Effect of Cu3BTC2 on membrane transport

properties. *J. Memb. Sci.* **428**, 331–340 (2013).

21. Wu, C. *et al.* Enhancing the Gas Separation Selectivity of Mixed-Matrix Membranes Using a Dual-Interfacial Engineering Approach. *J. Am. Chem. Soc.* **142**, 18503–18512 (2020).
22. Chen, G. *et al.* M-gallate MOF/6FDA-polyimide mixed-matrix membranes for C2H4/C2H6 separation. *J. Memb. Sci.* **620**, 118852 (2021).
23. Díaz, K., López-González, M., del Castillo, L. F. & Riande, E. Effect of zeolitic imidazolate frameworks on the gas transport performance of ZIF8-poly(1,4-phenylene ether-ether-sulfone) hybrid membranes. *J. Memb. Sci.* **383**, 206–213 (2011).
24. Bux, H., Chmelik, C., Krishna, R. & Caro, J. Ethene/ethane separation by the MOF membrane ZIF-8: Molecular correlation of permeation, adsorption, diffusion. *J. Memb. Sci.* **369**, 284–289 (2011).
25. James, J. B., Wang, J., Meng, L. & Lin, Y. S. ZIF-8 Membrane Ethylene/Ethane Transport Characteristics in Single and Binary Gas Mixtures. *Ind. Eng. Chem. Res.* **56**, 7567–7575 (2017).
26. Valadez Sánchez, E. P. *et al.* α-Al2O3-supported ZIF-8 SURMOF membranes: Diffusion mechanism of ethene/ethane mixtures and gas separation performance. *J. Memb. Sci.* **594**, 117421 (2020).
27. Japip, S., Liao, K.-S., Xiao, Y. & Chung, T.-S. Enhancement of molecular-sieving properties by constructing surface nano-metric layer via vapor cross-linking. *J. Memb. Sci.* **497**, 248–258 (2016).
28. Wijenayake, S. N. *et al.* Surface Cross-Linking of ZIF-8/Polyimide Mixed Matrix Membranes (MMMs) for Gas Separation. *Ind. Eng. Chem. Res.* **52**, 6991–7001 (2013).
29. Diestel, L., Wang, N., Schulz, A., Steinbach, F. & Caro, J. Matrimid-Based Mixed Matrix Membranes: Interpretation and Correlation of Experimental Findings for Zeolitic

Imidazolate Frameworks as Fillers in H2/CO2 Separation. *Ind. Eng. Chem. Res.* **54**, 1103–1112 (2015).

30. Campo, M. C., Magalhães, F. D. & Mendes, A. Carbon molecular sieve membranes from cellophane paper. *J. Memb. Sci.* **350**, 180–188 (2010).
31. Hatori, H., Takagi, H. & Yamada, Y. Gas separation properties of molecular sieving carbon membranes with nanopore channels. *Carbon N. Y.* **42**, 1169–1173 (2004).
32. Omidvar, M. *et al.* Unexpectedly Strong Size-Sieving Ability in Carbonized Polybenzimidazole for Membrane H2/CO2 Separation. *ACS Appl. Mater. Interfaces* **11**, 47365–47372 (2019).
33. Zhang, C. & Koros, W. J. Ultraselective Carbon Molecular Sieve Membranes with Tailored Synergistic Sorption Selective Properties. *Adv. Mater.* **29**, (2017).
34. Liang, J. *et al.* Effects on Carbon Molecular Sieve Membrane Properties for a Precursor Polyimide with Simultaneous Flatness and Contortion in the Repeat Unit. *ChemSusChem* **13**, 5531–5538 (2020).
35. Yoshimune, M., Fujiwara, I., Suda, H. & Haraya, K. Novel Carbon Molecular Sieve Membranes Derived from Poly(phenylene oxide) and Its Derivatives for Gas Separation. *Chem. Lett.* **34**, 958–959 (2005).
36. Lei, L. *et al.* Carbon hollow fiber membranes for a molecular sieve with precise-cutoff ultramicropores for superior hydrogen separation. *Nat. Commun.* **12**, 268 (2021).
37. Japip, S., Liao, K.-S. & Chung, T.-S. Molecularly tuned free volume of vapor cross-linked 6FDA-Durene/ZIF-71 MMMs for H2 /CO2 separation at 150 °C. *Adv. Mater.* **29**, (2017).
38. Yang, T., Xiao, Y. & Chung, T.-S. Poly-/metal- benzimidazole nano-composite membranes for hydrogen purification. *Energy Environ. Sci.* **4**, 4171–4180 (2011).
39. Yang, T., Shi, G. M. & Chung, T.-S. Symmetric and asymmetric zeolitic imidazolate

frameworks (ZIFs)/polybenzimidazole (PBI) nanocomposite membranes for hydrogen purification at high temperatures. *Adv. Energy Mater.* **2**, 1358–1367 (2012).

40. Bux, H. *et al.* Oriented Zeolitic Imidazolate Framework-8 Membrane with Sharp H2/C3H8 Molecular Sieve Separation. *Chem. Mater.* **23**, 2262–2269 (2011).

41. Bux, H. *et al.* Zeolitic imidazolate framework membrane with molecular sieving properties by microwave-assisted solvothermal synthesis. *J. Am. Chem. Soc.* **131**, 16000–16001 (2009).
